# Editorial – Synthetic Biology: Engineering Complexity and Refactoring Cell Capabilities

**DOI:** 10.3389/fbioe.2015.00120

**Published:** 2015-08-21

**Authors:** Francesca Ceroni, Pablo Carbonell, Jean-Marie François, Karmella A. Haynes

**Affiliations:** ^1^Centre for Synthetic Biology and Innovation, Imperial College London, London, UK; ^2^Department of Bioengineering, Imperial College London, London, UK; ^3^SYNBIOCHEM, Manchester Institute of Biotechnology, Faculty of Life Sciences, University of Manchester, Manchester, UK; ^4^LISBP, INSA, INP, UPS, Université de Toulouse, Toulouse, France; ^5^MR792, Ingénierie des Systèmes Biologiques et des Bioprocédés, INRA, Toulouse, France; ^6^UMR 5504, CNRS, Toulouse, France; ^7^Ira A. Fulton School of Biological and Health Systems Engineering, Arizona State University, Tempe, AZ, USA

**Keywords:** synthetic biology, complexity, metabolic engineering, emerging properties, crosstalk

Synthetic Biology is now in its second decade and many goals have been achieved toward the rational design of biological systems. This Research Topic features and reviews some of the latest progress in Synthetic Biology with a focus on research at the intersection between rational design and natural complexity with a potential outcome to concrete biotechnological applications. Kelwick et al. (2014) summarize the great expansion in the genetic toolkit and DNA assembly techniques that are currently available for synthetic biologists. These tools will advance the implementation of new functions and the production of useful metabolites in living cells in a controlled fashion. Using engineering formality, Synthetic Biology aims to identify biological design principles that can be used for practical applications. As one of the results, metabolic engineering is now becoming feasible to introduce novel functions and properties into an increasing number of microbial hosts. Examples come from Yu et al. (2014) and Heider et al. (2014) that describe the production of fatty-acid-derived chemicals and astaxanthin in microbes, respectively. Furthermore, bacteria can be engineered for the conversion of waste into renewable products, as Nieves et al. (2015) demonstrate with the bioconversion of lignocellulose.

Along with its great successes, Synthetic Biology is also encountering new challenges, represented by emerging behaviors in modified host cells (chassis) that are difficult to predict. Limitations in the robust prediction of gene networks arise from the lack of a proper understanding of the living systems used in synthetic biology. For instance, Akhtar and Jones (2014) appropriately present the evidence that the failure of a number of pathway engineering strategies are often due the lack of co-factors needed for the proper activity of the key enzymes. Co-factor production needs to be integrated in the system’s design to achieve proper enzymatic activity. As synthetic network designs become more complex, emerging evidence shows that elements within these networks can exhibit crosstalk and lead to non-specific behavior. As presented in Davis et al. (2015), bacterial quorum sensing pathways, which are widely used in Synthetic Biology, exhibit crosstalk that can limit the number of nodes in a network, and therefore stifle efforts to build sophisticated systems. New efforts are needed to better understand the behavior of composable parts and to develop new orthogonal elements. Lastly, Beal (2015) addresses unresolved questions in the area of cell-based information processors and noise. He proposes a quantitative signal-to-noise ratio-based standard to assess circuit performance.

An important aspect in the engineering of living cells that only recently has been investigated in detail by the synthetic biology community is the interaction between the system and the chassis. The exploitation of the cell’s resources for the operation of heterologous systems has proven to be deleterious, leading to non-robust gene expression and inefficient cellular performance with decreased population growth. In that direction, Moya (2014) reflects on the need of controllable systems in synthetic entities preventing obsolescence, similarly as how living cells exhibit self-maintenance. In Fehér et al. (2015) the observation of the dynamic response of a malonyl-CoA biosensor in *Escherichia coli* was used to understand the toxicity of the overproduction of a synthetic compound, which interfered with the system’s behavior. Martínez-García et al. (2014) present the development of new broad host range Tn5 vectors in order to relieve the burden of PHB production on the health of gram negative bacteria (*E. coli*). These studies address the need to investigate and develop controlled production of the molecule of interest to avoid burden-related negative feedback from the chassis.

As illustrated by the works in this Research Topic, now is a critical moment for Synthetic Biology, where the initial enthusiasm for the major achievements attained gives way to a deeper and better understanding of the complexity of biological systems. Advancing in this direction will significantly improve the applicability of design principles for living organisms.

## Conflict of Interest Statement

The authors declare that the research was conducted in the absence of any commercial or financial relationships that could be construed as a potential conflict of interest.
